# Histotripsy for Liver Malignancies: A Systematic Review of the Existing Literature

**DOI:** 10.1007/s12029-026-01632-6

**Published:** 2026-11-14

**Authors:** Morgan E. Roberts, Natalie M. Bath

**Affiliations:** Department of Surgery, University of South Alabama Health University Hospital, 2451 University Hospital Drive, Mastin 705, Mobile, AL 36617 USA

**Keywords:** Histotripsy, Liver directed therapies, Liver neoplasms, Hepatic metastases, Ablation, Acoustic cavitation

## Abstract

**Background:**

Histotripsy is an emerging noninvasive, nonthermal ablative therapy recently introduced for the treatment of hepatic malignancies. This systematic review evaluated the efficacy and safety of histotripsy for primary and secondary liver tumors.

**Methods:**

PubMed and Scopus were searched from inception through August 2026 in accordance with PRISMA guidelines. Search terms included combinations of subject headings and keywords such as “histotripsy,” “hepatic,” and “metastases.”

**Results:**

Ten clinical studies published between 2022 and 2026 met inclusion criteria. Study populations included hepatocellular carcinoma and metastases from colorectal, neuroendocrine, pancreatic, breast, and other malignancies. Technical success ranged from 68.8% to 100%, although definitions varied across studies. Early treatment efficacy and local control were generally high among successfully treated tumors, with several studies reporting rates exceeding 90%. Longer-term oncologic data were limited by heterogeneous imaging criteria, treatment intent, and patient populations. Major complications were uncommon, although vascular thrombosis and acute kidney injury emerged as potential safety concerns.

**Conclusions:**

Current evidence supports the technical feasibility and short-term efficacy of hepatic histotripsy. However, standardized outcome definitions, longer follow-up, and comparative studies are needed to establish its durability and role relative to existing liver-directed therapies.

**Supplementary Information:**

The online version contains supplementary material available at https://doi.org/10.1007/s12029-026-01632-6.

## Introduction

Liver malignancies, including both primary liver tumors and metastatic disease, remain a substantial source of cancer-related morbidity and mortality worldwide [1]. Liver metastases portend a poor prognosis, with significantly lower one-year survival reported among patients with synchronous liver metastases when compared to patients with non-hepatic metastatic disease [2].

Although complete surgical resection remains the gold standard for management of liver malignancies in select populations, many patients, particularly those with liver metastases, are not candidates for resection due to tumor location or burden, insufficient hepatic reserve, medical comorbidities, or extrahepatic disease [3–5]. Previous studies have shown fewer than 30% of patients with hepatocellular carcinoma (HCC) and approximately 20% of patients with colorectal liver metastases are deemed appropriate surgical candidates at presentation [5, 6]. Furthermore, despite advances in operative techniques and perioperative care, liver resection remains associated with substantial morbidity [4].

As a result, locoregional and ablative therapies, including transarterial chemoembolization (TACE), microwave ablation (MWA), radiofrequency ablation (RFA), hepatic arterial infusion chemotherapy (HAIC), and high-intensity focused ultrasound (HIFU) have become increasingly popular management strategies, serving as both definitive and adjunctive modalities among patients with unresectable disease [3, 7]. However, despite their evolving clinical utility, several limitations of locoregional therapies remain including decreased efficacy with increasing tumor size and limited ability to treat lesions adjacent to major vascular or biliary structures due to heat-sink effects or the risk of thermal injury to surrounding tissues. Other observed complications include bleeding, bile duct injury, and hepatic decompensation [7–15]. These limitations have created interest in ablative therapies capable of achieving local tumor destruction without relying on thermal energy.

Initially developed at the University of Michigan in 2004, histotripsy is a non-invasive, non-thermal, non-ionizing, ultrasound-based technology that received US Food and Drug Administration (FDA) approval for the treatment of primary and secondary liver tumors in October 2023 [14–17]. The delivery of short-duration, high-amplitude ultrasound pulsations at a low duty cycle results in acoustic cavitation, causing mechanical disruption of the targeted tissue at a cellular level [16, 17]. In contrast to conventional thermal ablative therapies which rely on the generation of heat to induce tissue destruction, histotripsy produces a mechanical strain that results in the breakdown of cellular structures [16, 18]. The purpose of this systematic review is to evaluate the current clinical evidence regarding the efficacy and safety of histotripsy for the treatment of liver metastases.

## Methods

### Study Design and Ethics

This systematic review was performed in accordance with the PRIMSA 2020 guidelines. Institutional review board approval was not required as all data was obtained from previously published studies.

### Eligibility Criteria

Eligibility criteria were developed in accordance with the population, intervention, comparison, and outcome (PICO) framework. English-language studies evaluating histotripsy as a treatment modality for liver metastases in adults (≥ 18 years) and reporting at least one clinically relevant outcome measure were considered eligible for inclusion.

Eligible study designs included randomized and non-randomized clinical trials, prospective and retrospective cohort studies, and case series. Studies evaluating benign liver disease, non-human or pre-clinical models, histotripsy for non-oncologic indications, or non-histotripsy focused ultrasound technologies were excluded. Editorials, commentaries, letters, reviews, case reports involving fewer than three patients, conference abstracts with insufficient data, duplicate publications, and studies without accessible full text were also excluded. When multiple publications reported outcomes from the same or substantially overlapping cohort, the more recent or comprehensive report was selected. Earlier publications were not counted separately in the final number of included studies but were reviewed when they provided unique or more detailed outcome data not reported in the subsequent publication.

### Search Strategy and Screening

A systematic search of the PubMed and Scopus databases was performed to identify articles published from database inception through August 2026.

The search terms used included various combinations of “histotripsy”, “acoustic cavitation”, “boiling”, “ultrasound”, “ablation”, “noninvasive”, “mechanical tissue ablation”, “high intensity focused ultrasound”, “HIFU”, “hepatic”, “liver”, “cancer”, “malignancy”, “metastasis”, and “tumor”. Detailed search formulas are listed in Supplementary Materials 1.

Additional studies were identified through manual review of reference lists of included studies and relevant systematic reviews. Because histotripsy is an emerging technology with a relatively limited clinical literature, a search of ClinicalTrials.gov was performed to identify ongoing or unpublished clinical trials. The final database search was performed on August 2, 2026.

Following the removal of duplicate records, all titles and abstracts were independently screened by two reviewers. Records considered potentially relevant by either reviewer underwent full-text assessment according to prespecified eligibility criteria. Discrepancies regarding study eligibility were resolved by thorough discussion or consultation with a third reviewer.

### Data Extraction

Extracted variables included study design, patient and tumor characteristics, treatment characteristics, and clinical outcomes. Outcomes of interest included technical success, tumor response, local tumor control, recurrence, survival, and treatment-related complications.

Several publications included known or suspected overlapping patient populations. Tumor histology and anatomic location were summarized as reported within each publication and were not adjusted for overlapping cohorts. As a result, tumor type and location counts may include duplicate representation and should not be interpreted as unique numbers of patients or lesions.

## Results

### Study Selection

**Fig. 1:**
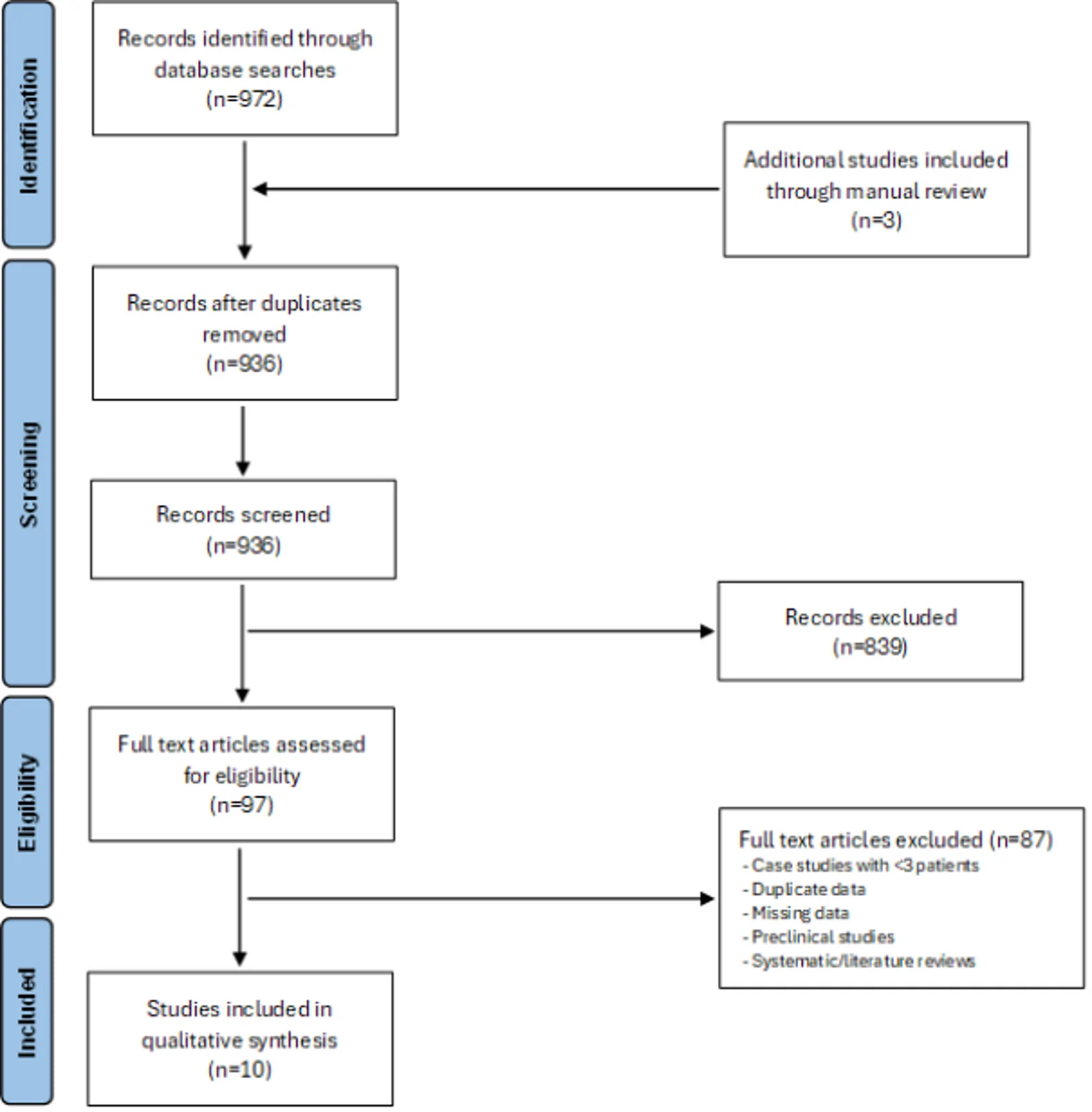
PRISMA Diagram for Search Strategy of Included Studies

The study selection process is demonstrated in the PRISMA flow diagram in Fig. 1. A total of 10 clinical studies met the predefined eligibility criteria and were included in the final systematic review [19–28]. Of note, the original #HOPE4LIVER trial and its subsequent one-year follow-up represent overlapping reports of the same cohort. To avoid duplicate representation, the one-year follow-up was retained as the primary report, and the original publication was not counted separately in the final number of included studies. The original publication was reviewed for outcomes that were not available in the one-year follow-up, including early technical efficacy and safety outcomes [29].

### Study Characteristics

The ten included studies were conducted across 5 countries and published between 2022 and 2026. Two studies were single-arm, non-randomized clinical trials and eight were observational cohort studies. Three studies were prospective and seven were retrospective.

Individual sample sizes ranged from 8 to 295 patients, with follow-up ranging from 30 days to 12 months. Because several publications included known or suspected overlap of patient populations, study-level sample sizes were not summed to estimate the total number of unique patients represented.

The study populations included patients with both primary and secondary liver malignancies. Reported tumor histologies included HCC (*n* = 89) and metastases from colorectal (*n* = 204), neuroendocrine (*n* = 97), pancreatic (*n* = 69), breast (*n* = 52), cholangiocarcinoma (*n* = 36), and other malignancies (*n* = 54). Colorectal liver metastases, neuroendocrine tumors, and HCC were the most frequently represented malignancies. These cumulative counts were not adjusted for overlapping cohorts and therefore, should not be interpreted as a unique patient count. Extrahepatic disease was inconsistently reported, although several studies included patients with concurrent extrahepatic metastases, with prevalence reaching 71.4% in one cohort [19].

Most cohorts consisted predominantly of older adults with preserved functional status. The median age exceeded 60 years in most studies, and women represented approximately 57% of reported patients. When reported, most patients had an ECOG performance status of 0 or 1 and Child-Pugh class A liver function. Many patients had also received systemic therapy, with prior systemic treatment exceeding 90% in some cohorts [22, 23].

Tumor and treatment characteristics varied across included studies. Mean reported tumor size ranged from 1.3 to 3.6 cm. Studies included patients with both solitary and multiple liver tumors, and the number of lesions treated differed substantially between cohorts and individual patients. Tumor location was distributed across multiple liver segments with the most commonly targeted lesions noted in segments III (*n* = 248), IV (*n* = 143), VI (*n* = 129), and V (*n* = 119). These segment-level counts were not adjusted for overlapping cohorts.

Treatment times varied according to tumor burden and volume treated. When reported, median treatment time per tumor ranged from approximately 13.5 to 34 min. Treatment intent also varied, with some studies distinguishing complete treatment from partial or palliative treatment, a distinction particularly relevant among patients with extensive metastatic disease or tumors in which complete treatment was not feasible. Detailed characteristics of the included studies are presented in Table 1.

**Table 1:** Characteristics of included studies evaluating histotripsy for hepatic malignancies

	Vidal-Jove et al., [19]	Wehrle et al., [26]	Ziemlewicz et al., [20]	Mabud et al., [23]	Mabud et al., [22]	Chan et al., [21]	Wehrle et al., [24]	Wehrle et al., [﻿^25^]	Jimenez-Soto et al., [28]	Liu et al., [27]
**Study Characteristics**										
Location	Spain	USA, UAE	USA, Europe	USA	USA	Hong Kong	USA	USA	USA	USA
Study design	Prospective, non-randomized multicenter trial	Retrospective, multicenter cohort study	Prospective, non-randomized, multicenter trial	Retrospective, single-center cohort study	Retrospective, single-center cohort study	Prospective, single-center cohort study	Retrospective, multicenter cohort study	Retrospective, single-center cohort study	Retrospective, single-center cohort study	Retrospective, single-center cohort study
Study period	07/18-05/19	12/23-07/24	01/21-12/22	02/24-01/25	02/24-01/25	08/24-12/24	12/23-12/24	12/23-11/24	12/23-01/25	06/24-10/24
Sample size	8	295 treated; 230 in safety analysis	47	21	28	30	47	71	27	27
**Patient Characteristics**										
Age, years	60.4 (52.6–74.7)	60 (58–67)	65 (36–89)	64.7 (±13.6)	59.1 (±15.6)	63.5 (43–88)	64 (51–74)	60.3 (47.4–69)	59 (±12.7)	NR
Sex, n (%)										
Male	3 (37.5)	94 (41)	24 (51.1)	13 (62)	13 (46)	17 (56.6)	NR	28 (39.4)	13 (48)	6 (22)
Female	5 (62.5)	136 (59)	23 (48.9)	8 (38)	15 (54)	13 (43.4)		43 (60.6)	14 (52)	21 (78)
Eligibility criteria	Not eligible for any other therapy	Underwent histotripsy after FDA approval	Not eligible for any other therapy	Stage IV PDAC with hepatic metastases	Liver metastases, not eligible for any other therapy	Primary or secondary liver lesion <10 cm	Received histotripsy with treatment intent of complete control	Received histotripsy for primary or secondary liver lesion	Patients who may benefit from non-invasive and non-thermal treatment	Stage IV NET with hepatic metastases and adequate US windows
**Tumor Histology, n (%)**										
HCC	1 (12)	31 (11)	19 (4)	0	0	19 (63)	7 (15)	7 (10)	5 (19)	0
Colorectal	5 (63)	117 (40)	11 (23)	0	9 (32)	4 (13)	13 (28)	28 (39)	17 (63)	0
Neuroendocrine	0	46 (16)	2 (4)	0	3 (11)	0	6 (13)	11 (15)	2 (7)	27 (100)
Pancreatic	0	30 (10)	5 (11)	21 (100)	0	3 (10)	6 (13)	4 (6)	0	0
Breast	1 (12)	26 (9)	4 (9)	0	5 (18)	1 (3)	6 (13)	8 (11)	1 (4)	0
Cholangiocarcinoma	1 (12)	23 (8)	0	0	4 (14)	2 (7)	2 (4)	4 (6)	0	0
Other	0	22 (7)	6 (13)	0	7 (25)	1 (3)	7 (15)	9 (13)	2 (7)	0
**Tumor Characteristics**										
Tumors treated, n	11	510	52	39	56	60	91	109	42	35
Tumor size, cm	1.4 (0.9–1.6)	2.85	1.3 (0.4–2.8)	2.65 (±1.25)	3.58 (±3.4)	1.8 (0.6–5.4)	2.55 (1.8–3)	2 (1.4–3.3)	3 (2.03–4.18)	NR
Hepatic Segment, n (%)										
I	0	9 (2)	0	0	0	2 (3)	NR	NR	4 (9)	NR
II	4 (36)	60 (12)	10 (19)	1 (3)	5 (9)	12 (20)		12 (11)	3 (7)	
III	4 (36)	140 (27)	27 (52)	10 (26)	16 (29)	21 (35)		25 (23)	5 (12)	
IV	0	99 (19)	9 (17)	3 (8)	7 (13)	4 (7)		16 (15)	5 (12)	
V	1 (9)	79 (15)	3 (6)	14 (36)	11 (20)	4 (7)		NR	7 (17)	
VI	0	98 (18)	3 (6)	4 (10)	6 (11)	9 (15)		7 (6)	4 (9)	
VII	0	29 (6)	0	0	4 (7)	2 (3)		NR	2 (5)	
VIII	0	8 (2)	0	1 (3)	0	6 (10)		NR	2 (5)	
Multiple	2 (18)	0	0	6 (15)	7 (13)	0		49 (45)	9 (21)	
Extrahepatic disease, n (%)	5 (71.4)	NR	14 (50)	NR	NR	NR	6 (13)	NR	8 (29)	NR
Prior systemic therapy, n (%)	NR	NR	16 (34)	20 (95)	27 (96)	NR	NR	14 (20)	NR	NR
Current systemic therapy, n (%)	NR	NR	NR	17 (81)	24 (86)	NR	38 (81)	47 (66)	20 (74)	NR
**Treatment Characteristics**										
Treatment intent, n (%)										
Complete	NR	NR	NR	16 (41)	28 (50)	NR	91 (100)	86 (79)	30 (71)	16 (46)
Partial				23 (59)	28 (50)		0	23 (21)	12 (29)	19 (54)
Median length of procedure, min	25 (15–30)	22.6	201 (107–420)	NR	NR	13.5 (4.2–60) *per tumor	22.6 (19.5–36.5) *per tumor	NR	NR	97–473 *range
Follow-up, months	2	1	12	1	3	1	3	3	3	4

*UAE* United Arab Emirates, *USA* United States of America, *NR* not recorded, *FDA* U.S. Food and Drug Administration, *HCC* hepatocellular carcinoma, *PDAC* pancreatic ductal adenocarcinoma, *NET* neuroendocrine tumor, *US* ultrasound

Technical success, treatment efficacy, and assessment of safety profile served as the primary and secondary outcomes for most of the included studies.

### Efficacy Outcomes

Technical success was generally high across studies, ranging from 68.8% to 100%. Definitions varied across studies and included successful delivery of the planned treatment, complete coverage of the target tumor by the treatment zone, and absence of residual viable tumor on early post-treatment imaging. The THERESA trial achieved its predefined acute technical success endpoint for all treated tumors [19], while the original #HOPE4LIVER trial achieved technical success in 42 of 44 (95%) tumors [29]. Similar technical success rates of 95% or greater were reported in several subsequent studies [21–23].

Technical success was less consistent when adequacy was determined based on early tumor viability rather than treatment delivery alone. In the first full year histotripsy experience described by Wehrle et al., 60 of 82 (73%) tumors intended for complete treatment were considered nonviable on postoperative day 1 imaging [25]. Similarly, a single-center study evaluating neuroendocrine liver metastases by Liu et al., technical success was achieved in 11 of 16 tumors treated with complete intent [27].

Early treatment efficacy and local tumor control were also favorable when complete treatment was technically achieved, with reported rates ranging from 82.5% to 100% across studies. Once again, definitions and imaging criteria differed substantially between studies. In the #HOPE4LIVER trial, technique efficacy was 83% at 30 days. Similarly, a single center prospective analysis performed by Chan et al. found a 30 day efficacy of 82.5% among successfully treated tumors [21]. A multicenter study of 91 tumors described by Wehrle et al. reported nonviability of 95% of treated tumors at 30 days and a primary efficacy rate of 94% at 90 days. Two tumors with residual viable disease underwent repeat histotripsy and subsequently achieved complete response, resulting in a secondary efficacy rate of 96% [24]. The neuroendocrine tumor cohort demonstrated a local tumor control rate of 100% among successfully treated tumors targeted with complete treatment intent. In contrast, tumors that were incompletely treated demonstrated partial response, stable disease, or progression, highlighting the distinct difference between technical treatment success and subsequent efficacy [27].

Longer-term data regarding the efficacy of histotripsy remain limited, as most studies primarily reported short-term outcomes, restricting assessment of durable local control and long-term oncologic benefit. The one-year #HOPE4LIVER update demonstrated complete treatment-zone coverage in 45 of 47 (95.7%) tumors. Using the predefined imaging assessment, local control was observed in 79.2%, 80%, and 91.7% of evaluated tumors at 30 days, 6 months, and 1 year, respectively. A post hoc assessment developed to account for the evolving interpretation of post-histotripsy imaging demonstrated absence of local progression in 89.6%, 91.7%, and 96% at the corresponding time points. The reported one-year local control rate was 63% using the primary assessment and 90% using the post hoc assessment. Median survival time was 20.7 months for all patients, with a higher one-year overall survival observed among those with HCC in comparison to patients with secondary liver metastases [20].

Outcomes were more variable in cohorts that included partial or palliative treatment intent. A study conducted by Jimenez-Soto et al. evaluated patients for up to one year with the intent of using histotripsy to either bridge to liver transplant or resection (*n* = 9) or for palliative purposes (*n* = 18). Local control was achieved in 10 of 17 patients (59%) at 3 months and 5 of 10 patients (50%) at 6 months. Notably, local control during the first 6 months was generally higher among those treated with bridging or downstaging intent than among those treated with palliative intent, reaching 87.5% (7/8) versus 33% (3/9) at 3 months and 100% (3/3) versus 29% (2/7) at 6 months, respectively. Ultimately, three of the four surgical candidates underwent hepatic resection, and all five potential transplant candidates were approved for liver transplantation. Beyond 6 months, imaging was available for very few patients, limiting further assessment of continued oncologic benefit [28]. Efficacy outcomes for the included studies are summarized in Table 2.

**Table 2:** Efficacy outcomes following histotripsy for hepatic malignancies

	Sample size	Tumors treated, *n*	Technical/initial treatment endpoint	*n*/*N* (%)	Early efficacy endpoint	*n*/*N* (%)	Longer-term efficacy
Vidal-Jove et al., [19]	8	11	Acute technical success on POD 1 MRI (creation of planned tissue destruction volume)	11/11 (100)	NR	‒‒	NR
Wehrle et al., [26]	295	510	NR	‒‒	NR	‒‒	NR
Ziemlewicz et al., [20]	47	52	Complete coverage by histotripsy treatment zone ≤36 h	45/47 (95.7)	Absence of nodular or mass-like enhancement at 30d	38/48 (79.2)	Primary assessment: 28/35 (80%) at 6mo, 22/24 (91.7%) at 1y Post hoc absence of local progression: 43/48 (89.6%) at 30 d, 33/36 (91.7%) at 6mo, 24/25 (96%) at 12mo
Mabud et al., [23]	21	39	Successful delivery of planned histotripsy treatment	39/39 (100)	Nonenhancing area of treatment among fully treated lesions at 1mo	9/9 (100)	7/8 (87.5%) showed no suspicious enhancement at 3mo 3 fully treated lesions with ≥6mo imaging demonstrated 85–95% volume reduction without suspicious enhancement
Mabud et al., [22]	28	56	Technical success	54/56 (97)	Fully treated tumors demonstrating hypoattenuation without nodular enhancement at 1mo	20/20 (100)	5/5 (100% of fully treated tumors demonstrated hypoattenuation without nodular enhancement at 3mo
Chan et al., [21]	30	60	Technical success at 36 h	57/60 (95)	Efficacy at 1 month among technically successful tumors	47/57 (82.5)	NR
Wehrle et al., [24]	47	91	NR	‒‒	Absence of viable tumor/primary efficacy at 30d	86/91 (95)	Primary efficacy at 90d: 80/85 (94%) Secondary efficacy after retreatment: 82/85 (96%)
Wehrle et al., [﻿^25^]	71	109	Tumor nonviability on POD 1 among lesions intended for complete treatment	60/82 (73)	Persistent nonviability at 30 d among tumors nonviable on POD 1	60/60 (100)	23/23 (100%) of evaluated tumors remained nonviable at 90d
Jimenez-Soto et al., [28]	27	42	NR	‒‒	Local control (stable or decreased lesion size) at 1-3mo	1mo: 6/8 (75) 3mo: 10/17 (59)	Local control rate of 50% (5/10) at 6mo, but outcomes notably varied by treatment intent 3/4 patients downstaged to resection, 5/5 patients approved for liver transplant
Liu et al., [27]	27	35	Technical success on first follow-up imaging among tumors treated with complete intent	11/16 (69)	Complete response/local control among technically successful tumors	11/11 (100)	All technically successful lesions maintained complete response during available follow up

*NR* not reported, *POD* postoperative day, *MRI* magnetic resonance imaging

### Safety Outcomes

Adverse event definitions and surveillance periods varied across studies. Many studies used the Clavien-Dindo grading system to classify complications with grades I-II categorized as minor adverse events and grade III or higher categorized as major adverse events. Because studies variably reported event counts and numbers of affected patients, an overall complication rate range was not calculated.

Overall, histotripsy was associated with low rates of major complications, with several studies reporting no major treatment-related adverse events [19, 21, 24]. The pivotal #HOPE4LIVER trial reported major complications in 3 of 44 patients (6.8%), while a large, multicenter safety study of 230 patients reported a major complication rate of only 1.3% with these events largely attributed to malignancy progression rather than histotripsy [26, 29].

Minor adverse events were more common and often self-limited, including postprocedural pain, fever or flu-like symptoms, and transient skin changes or irritation. Vascular complications, namely thrombosis, were reported in several studies, with severity varying based on location. Portal vein thrombosis was reported most commonly (*n* = 13) with hepatic vein thrombosis seen less frequently (*n* = 1). Additional reported complications included bacteremia, pneumonia, and in one case, a biloma. When assessed, only a single complication was reported to occur outside of the initial 30-day post-treatment window. Most cohorts reported short hospital stays, with several demonstrating same-day or next-day discharges. Of note, the study evaluating patients with neuroendocrine liver metastases reported three cases of acute kidney injury (AKI) among a cohort of only 27 patients, with one patient requiring hemodialysis. The authors proposed tumor histolysis as a potential contributor to AKI [27].

Treatment related mortality was uncommon, and deaths reported during follow-up were largely attributed to progression of underlying malignancy or medical comorbidities rather than the procedure.

Overall, the short-term safety profiles were favorable, however, differences in patient populations, treatment extent, and in some cases, definitions of adverse events limited direct comparison across studies. Safety outcomes are summarized in Table 3.

**Table 3:** Safety outcomes following histotripsy for hepatic malignancies

	Safety population, *n*	Total adverse events, *n*	Minor complications, *n*	Major complications, *n*	Major complications reported	Mortality
Vidal-Jove et al., 2022 [﻿^19^]	8	0	0	0	None	0
Wehrle et al., [26]	230	12	9	3	Death due to malignancy progression^¥^	3 deaths^¥^
Ziemlewicz et al., [20]	47	49	43	6	Hepatic failure, PVT, thrombosis, procedural pain, sepsis, pleuritic pain	1 device-related death at ≤90d
Mabud et al., [23]	21	2	2	0	None	0
Mabud et al., [22]	26	4	3	1	Bacteremia	0 at ≤30d
Chan et al., [21]	30	7	7	0	None	0
Wehrle et al., [24]	47	3	3	0	None	0
Wehrle et al., [26]	71	35	34	1	Right hepatic vein thrombus	0 treatment-related
Jimenez-Soto et al., [28]	27	14	11	3ǂ	Septic shock/pneumonia (1), progression of liver disease (1), surgery required for progressive disease (1)	0 at ≤30d
Liu et al., [27]	27	57	52	5	AKI (3), respiratory distress/death (1), disease progression/death (1)	1 early treatment-associated death

*PVT* portal vein thrombosis, *AKI* acute kidney injury

¥ The 3 grade V events were deaths from progression of advanced malignancy and were considered unrelated to histotripsy by the authors

ǂ Major events occurred in patients with advanced/progressive disease and were not necessarily attributable to histotripsy

### Ongoing Clinical Trials

Seven ongoing clinical studies evaluating the use of histotripsy for liver tumors were identified, with planned enrollments ranging from 12 to 5,000 participants [30–36]. These studies include prospective interventional trials and observational registries assessing longer-term efficacy, specific tumor populations, challenging anatomic locations, and potential systemic effects.

The BOOMBOX Master Study (NCT06486454) is the largest, with a planned enrollment of 5,000 patients and follow up extending to five years [36]. Other studies are evaluating six-month local control (NCT07428356), tumors up to 10 cm (NCT06579833), lesions adjacent to major intrahepatic vessels (NCT07063771), and patients with unresectable symptomatic liver tumors (NCT07180706) [30–32, 34]. Disease-specific investigations include a phase II study of histotripsy combined with chemotherapy for unresectable, liver-confined colorectal metastases (NCT07044362) and the ECHO-CRC trial evaluating circulating immune markers and T-cell responses following histotripsy (NCT07361107) [33, 35].

Collectively, these studies represent a shift from initial feasibility and safety assessment toward longer-term efficacy, disease-specific applications, multimodal therapy, and potential systemic biologic effects.

Summarized characteristics of the ongoing clinical studies are presented in Table 4.

**Table 4:** Ongoing studies evaluating histotripsy for primary and secondary liver malignancies

Title (ClinicalTrials.gov Identifier)	Location	Recruitment status	Estimated completion date	Study design	Sample size	Primary outcome(s)	Secondary outcome(s)
Histotripsy (HistoSonics^®^) for Liver Tumors: a Phase II Safety and Efficacy Study (NCT06579833)	Hong Kong	Active, not recruiting	September 2028	Phase II, open-label, single-arm, single-center	60	Changes in tumor features Adverse event rate	N/A
Evaluation of Circulating Immune Response After Histosonics in Colorectal Cancer (ECHO-CRC) (NCT07361107)	United States	Recruiting	September 2027	Phase I, open-label, single-arm, single-center	12	Measure of systemic immune modulation via T cells	Kinetics of peripheral immune cell response via T cells
To Evaluate the Safety and Efficacy of Histotripsy for the Symptom Relief of Inoperable Abdominal Tumors (NCT07180706)	Taiwan	Not yet recruiting	July 2027	Open-label, single-arm, single-center	30	Symptom relief and quality of life	Tumor volume before and after treatment
Histotripsy Ablation for Liver Tumors (HALT): A Multi-centre Prospective Pilot Study on the Safety and Efficacy of Histotripsy in Asian Primary and Secondary Liver Malignancies (NCT07428356)	Singapore	Recruiting	May 2029	Phase I, open-label, single-arm, multicenter	40	Local control rate at 6 months Major complication rate	Technical success rate at 36 h Technique efficacy rate at 30 days
Safety and Clinical Efficacy of Histotripsy for Liver Cancers Adjacent to Major Intrahepatic Vessels (NCT07063771)	Taiwan	Not yet recruiting	July 2027	Phase I, open-label, single-arm, single-center	20	Symptom relief and quality of life	Tumor volume before and after treatment
Real-world Evaluation of the HistoSonics Edison System for Treatment of Liver Tumors Across Multidisciplinary Users (BOOMBOX: Master Study) (NCT06486454)	United States	Recruiting	November 2039	Open-label, single-arm, multicenter	5000	Technical success	N/A
Histotripsy Plus Chemotherapy for Advanced Colorectal Liver Metastasis: A Prospective, Single-Armed Trial (NCT07044362)	United States	Recruiting	October 2028	Phase II, open-label, single-arm, single-center	100	Radiologic tumor viability at 90 days	Rate of tumor necrosis and infiltration of immune cells (via biopsy) Overall and progression free survival Complication rate at 30 and 90 days

*N/A* non-applicable

## Discussion

### Summary of Key Findings

This systematic review summarizes the expanding clinical evidence for histotripsy use in liver metastases. Across the included studies, histotripsy demonstrated high rates of successful treatment delivery and early local tumor control with relatively low rates of major morbidity. Technical success was consistently high, exceeding 95% in several cohorts, and local control was particularly favorable when complete treatment was achieved. However, the evidence remains limited by predominantly single-arm study designs, heterogeneous tumor types and treatment intent, variable outcome definitions, and short follow-up. While the current literature supports the technical feasibility and short-term efficacy of histotripsy, it is not yet sufficient to establish oncologic superiority when compared to established treatments.

### Interpretation of Results

Technical success rates were generally high but not uniform across studies. Despite heterogeneous populations that frequently included heavily pretreated patients with substantial hepatic tumor burden or extrahepatic disease, several studies reported technical success rates between 95% and 100%, using definitions based on successful treatment delivery or complete tumor coverage. These rates are comparable to those reported for established thermal ablation techniques, with MWA technical success rates of approximately 92%−100% and complete ablation rates approaching 97% in randomized studies comparing MWA and RFA for early-stage HCC [37–39]. No included study directly compared histotripsy with thermal ablation, therefore, these comparisons should be interpreted cautiously because of differences in tumor characteristics, patient selection, and endpoint definitions.

Real-world experience has also identified technical limitations that were less apparent in earlier trials. In the first full year institutional cohort study, only 74% of tumors intended for complete treatment were nonviable on postoperative day 1, with tumor location, rib interference, bowel gas, and inadequate sonographic windows identified as potential barriers to targeting [25]. The neuroendocrine tumor cohort similarly achieved technical success in only 11 of 16 tumors treated with complete intent [27]. Notably, lesions in segments III, IV, V, and VI were most frequently represented across the included studies, which may partly reflect greater acoustic accessibility. These findings indicate that technical success may depend substantially on tumor location and accessibility in addition to treatment planning and operator experience. Furthermore, the non-invasive nature of histotripsy introduces a distinct set of technical constraints related to ultrasound visualization and acoustic access.

Early efficacy outcomes are encouraging, with most studies reporting high rates of treatment efficacy or local control within the first 30 to 90 days. For example, a multicenter study described by Wehrle et al. reported tumor nonviability in 95% of treated lesions at 30 days and a primary efficacy rate of 94% at 90 days [24]. Similarly, the #HOPE4LIVER trial and a prospective analysis performed by Chan et al. demonstrated favorable early treatment efficacy [21, 29].

Importantly, technical success should not be considered synonymous with oncologic efficacy. Several studies demonstrated that when complete treatment was achieved, subsequent local control was high. This was particularly evident in the neuroendocrine cohort, where technical success was achieved in only 11 of 16 tumors treated with complete intent, however, all 11 successfully treated tumors maintained complete response during the available follow-up period [27]. These observations suggest that adequate initial tumor treatment may be an important determinant of subsequent local control. At present, this conclusion is supported primarily by imaging-based outcomes and the number of patients with longer-term surveillance remains limited.

Longer-term oncologic outcomes are considerably less established. The one-year #HOPE4LIVER update provides some of the strongest available longitudinal evidence, with complete treatment-zone coverage demonstrated in 95.7% of tumors shortly after the procedure and consistently favorable radiographic findings through one year follow-up. Interpretation of these findings, however, is complicated by the evolving understanding of post-histotripsy imaging. The substantial difference between the prespecified and post hoc underscores the need for standardized, histotripsy-specific imaging criteria before outcomes can be reliably compared across studies [20].

Histotripsy has also been used beyond definitive local ablation, including for bridging or downstaging, palliation, and treatment of patients considered unsuitable for conventional liver-directed therapy. In the first FDA-approved.

institutional experience, local control was higher among patients treated with bridging or downstaging intent than among those treated for palliative purposes, and several patients ultimately underwent surgical resection or became eligible for transplant [28]. These findings require careful interpretation because treatment intent differed substantially between groups. Residual viable tumor following intentional partial or palliative treatment should not be considered equivalent to failure following complete-intent ablation. Future studies should therefore stratify outcomes according to treatment intent and planned treatment extent.

Histotripsy’s non-thermal mechanism may offer potential advantages for anatomically challenging tumors. Unlike thermal ablation, histotripsy does not depend on heat generation and therefore is not subject to the same heat-sink effect from adjacent bloody flow [11, 40, 41]. Several included studies reported treatment of lesions near major vascular structures, supporting further investigation of histotripsy use in locations that may be challenging for conventional thermal ablation. Whether this theoretical advantage translates into superior safety or local control has not yet been established.

The short-term safety profile was generally favorable, with several studies reporting no major treatment-related complications. Minor events were typically transient and included abdominal pain, fever, and skin changes. Several studies also reported short post-procedure hospital stays and same or next-day discharge, further suggesting a relatively limited procedural morbidity. These findings are particularly notable given that many treated patients had advanced malignancy and substantial prior treatment exposure.

Nevertheless, complications that warrant continued surveillance have been identified. Several studies reported findings of portal vein, and less frequently, hepatic vein, thrombosis. The recurrence of thrombotic events across separate clinical experiences suggests that vascular complications should be monitored as histotripsy use expands, particularly when treating tumors near major vessels. AKI may represent another potential treatment-specific complication. Liu et al. reported three cases of AKI among 27 patients undergoing histotripsy for neuroendocrine liver metastases and proposed extensive tumor histolysis as a potential mechanism [27]. More recently, a study described by Poe et al. reported AKIs following 9 of 146 hepatic histotripsy procedures (6.2%), with large and overlapping treatment volumes identified as possible risk factors [42]. Together, these reports raise concern that greater treatment volume and the extent of tissue destruction may contribute to post-histotripsy renal injury.

An additional area of interest is the possibility of systemic effects following histotripsy [43]. Reductions in untreated lesions have been described in several cohorts, including the first full year experience, in which decreases in untreated tumors were observed in select patients without changes in systemic therapy [25]. Similar observations were reported in the liver metastasis cohort, although recent or concurrent systemic therapy complicated the interpretation in these patients [22]. These observations are preliminary and should not be considered evidence of an established abscopal effect. Further prospective studies incorporating immune-related endpoints and controlling for concomitant systemic therapy are needed to determine the presence of a clinically meaningful systemic antitumor response.

### Comparison of Findings to Existing Literature

The findings of this review are broadly consistent with previous systematic reviews of hepatic histotripsy, which similarly reported high technical success and relatively low rates of major complications while identifying heterogeneous populations and limited follow-up as important weaknesses [44, 45]. The present review incorporates more recent evidence and further highlights limitations that have become apparent with expanding clinical use, including variable technical success in anatomically challenging tumors, evolving interpretation of post-treatment imaging, vascular complications, and AKI. Collectively, the expanding literature suggests that histotripsy has progressed beyond initial feasibility testing, although comparative effectiveness remains uncertain.

Several ongoing clinical studies may address important limitations of the current evidence. The BOOMBOX registry is expected to provide a substantial increase in the volume of real-world data, with follow-up extending to five years. Additionally, ongoing disease-specific trials may better define outcomes within more homogeneous patient populations. Studies evaluating colorectal liver metastases are particularly relevant and include investigation of histotripsy in combination with systemic chemotherapy and assessment of pathologic response following treatment, which has yet to be established and may help determine how reliably post-treatment imaging corresponds to tumor destruction. Studies investigating systemic immune responses following histotripsy may also help to clarify whether the biologic effects of treatment extend beyond local mechanical destruction.

### Strengths and Limitations

This review has several strengths, including an updated assessment of the rapidly expanding clinical literature, inclusion of both prospective and real-world evidence, evaluation of ongoing clinical trials, and separate consideration of technical success, subsequent treatment efficacy, and safety. Particular attention was also given to overlapping study populations to avoid inappropriate aggregation of repeated cohorts.

Several limitations must also be considered. The available literature predominantly consists of small, non-randomized, single-arm studies without comparison groups, limiting direct comparison of histotripsy with established liver-directed therapies.

Patient populations were heterogeneous with respect to tumor histology, disease burden, extrahepatic disease, and prior treatment, and treatment intent. Concurrent systemic therapy further obscures clinical interpretation. In one institutional series, 86% of patients remained on systemic therapy at the time of histotripsy. As a result, longer-term outcomes such as progression-free and overall survival cannot be attributed to histotripsy alone.

Outcome assessment was similarly heterogeneous. Follow-up was generally short, definitions of technical success and local tumor control were inconsistent, and efficacy was predominantly assessed radiographically rather than through pathologic confirmation. Histotripsy-specific imaging criteria have not yet been standardized, and the number of patients with available surveillance imaging declined substantially at later time points, introducing potential follow-up bias and limiting assessment of durability.

Lastly, differences in adverse event definitions and surveillance periods further limited comparison of safety outcomes, particularly because studies variably reported numbers of events or numbers of affected patients and did not always clearly distinguish complications attributable to histotripsy from those related to advanced malignancy.

These limitations emphasize the need for prospective studies using standardized outcome definitions, consistent imaging protocols, longer follow-up, and appropriate comparison groups to more definitively establish the safety, durability, and effectiveness of histotripsy relative to established liver-directed therapies.

## Conclusion

Histotripsy represents a promising addition to the growing collection of liver-directed therapies for liver metastases. Current evidence supports its technical feasibility and ability to early achieve local tumor control with relatively low short-term morbidity, particularly when complete treatment is technically achieved. At present, the existing literature is insufficient to determine its effectiveness when compared to other treatment modalities or its long-term oncologic benefit. Prospective studies with standardized outcome definitions, longer follow-up, and direct comparisons with established therapies are needed to define the appropriate role of histotripsy within multidisciplinary treatment of liver malignancies.

## Supplementary Information

Below is the link to the electronic supplementary material.

Supplementary Material 1.

## Data Availability

No datasets were generated or analysed during the current study.
